# Advances in Glycation: from food to human health and disease

**DOI:** 10.1007/s10719-021-09981-z

**Published:** 2021-03-19

**Authors:** Naoyuki Taniguchi, Masatsune Murata

**Affiliations:** 1 Department of Glyco-Oncology and Medical Biochemistry, Osaka International Cancer Institute, 3-1-69 Otemae, Chuo-ku, 541-8567 Osaka, Japan; 2 Department of Nutrition and Food Science, Ochanomizu University, 2-1-1 Otsuka, Bunkyo-ku, Tokyo, 112-8610 Japan

**Keywords:** Maillard reaction, Glycation of food, Glycation in disease, Advanced Glycation Endproducts (AGEs), Pigmentation, Browning, International Maillard Reaction Society (IMARS), Japan Maillard Reaction Society (JMARS)

## Abstract

This Special Issue on “Advances in Glycation: from food to human health and disease” was planned after the XXV International Symposium on Glycoconjugates (Glyco25) in Milan in order to ask special attention of importance of glycation to glycoscience community. In addition, we also celebrate the 30th anniversary of JMARS (Japan Maillard Reaction Society), and dedicated to one of the pioneers of this field, Professor Vincent Monnier, MD. He contributed enormously to studies on glycation related to aging and diseases to date and also he contributed to establish IMARS (International Maillard Reaction Society) as well as JMARS.

This special issue entitled “Advances in Glycation: from Food to Human Health and Diseases “was planned to appear after the XXV International Symposium on Glycoconjugates (Glyco25) in Milan in 2019, which was organized by Sandro Sonnino (University of Milan, chief editor of the Glycoconjugate J.) [1]. After the meeting was over, we proposed to publish a special issue focusing on glycation studies mainly taken from the speakers who attended this meeting.

When the 12th International Maillard Reaction Society (IMARS) was held in Tokyo in 2015, organized by the Japan Maillard Reaction Society (JMARS), a special issue entitled “Advanced glycation in diabetes, aging and age-related diseases: editorial and dedication “as published in the Glycoconjugate. J by Vincent Monnier and Naoyuki Taniguchi [2].

Sonnino also agreed with our proposal, and he also emphasized the importance of glycation research in Glycoconjugate research. Based on a survey of PubMed, the number of published papers related to glycation and Advanced Glycation Endproducts (AGEs) has increased dramatically in the past ten-odd years, as shown in the Fig. 1.

**Fig. 1:**
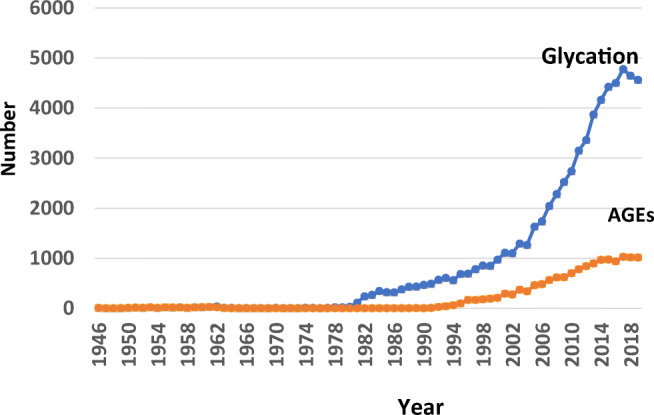
Increase of published papers on glycation and Advanced Glycation Endproducts (AGEs) calculated from PubMed

It is now well known that the Maillard reaction was first reported by Louis Camile Maillard at the Sorbonne University in 1912 [3]. In this report, he reported on the reaction products that are produced when aqueous solutions of reducing sugars turn progressively yellow-brown when heated or when stored under physiological conditions. Even though glycation, i.e., the Maillard reaction or the browning reaction or the amino-carbonyl reaction are quite common words in the field of food science and agricultural biochemistry, most people who are studying enzymatic glycosylation are not so familiar with this terminology. Moreover, the community of scientists who are involved in research regarding the Maillard reaction is rather small and drawing the attention of other members of the glycoconjugate community to this subject is important.

In medical science, since hemoglobin A1c was found to be a biomarker for serum glucose levels in diabetes mellitus [4] and moreover, excellent research on the Amadori rearrangement by John E. Hodge [5] and on advanced glycation end products (AGE) in aging and diabetes by Monnier and Anthony Cerami [6], Michael Brownlee [7], and John Baynes and Suzanne Thorpe [8], Antoinette Pirie [9]. Helen Vlassara [10] and many others also have caused this to attract the interest of members of the biomedical research community.

In addition, in Japan we had a long history of research on the browning reaction in food science because various Japanese fermented food items such as *Miso*, *Shoyu* and *Su* (rice vinegar) undergo browning reactions under maturation. The pioneering studies on the Maillard reaction in food science include research by Masao Fujimaki (University of Tokyo), Michio Namiki (Nagoya University) and Masamichi Kato (University of Tokyo) [11] and their colleagues. Several scientists from Japan spent time working with Monnier (Fumitaka Hayase, Satoshi Miyata, Motoko Takahashi, Shinji Taneda, Yoko Nishikawa, Masao Satake), Cerami (Zenji Makita and Masanobu Kawakami) and Baynes (Ryoji Nagai) as postdocs, and after they came back to Japan, they have continued their studies in academia or private clinics on the importance of glycation in medical science.

In 1989 Taniguchi (at that time at the Osaka University Medical School) discussed the Maillard reaction with Kato and Hidetaka Nakayama (a diabetologist at Hokkaido University) and launched the first research meeting on glycated proteins. This meeting has been held every year since then, even though the meeting name has been changed to JMARS. Therefore, in 2019 we hosted the 30th memorial meeting and, because of this, we felt that this would be a good occasion to publish a special issue.

Several distinguished scientists had been invited make presentations in the glycation session at the Glyco25 meeting and they gave excellent presentations. Among the International Maillard Society (IMARS) members, Vincent Monnier, a pioneer in this field, Paul Thornalley (Harmad Bin Khalifa University,), the president of IMARS、and Naira Rabbani (University of Warwick) were attendees, and from JMARS, the secretary general of JMAS, Yasuhiko Yamamoto (Kanazawa University) and Rhoji Nagai (Kumamoto University) were present along with several internationally known scientists who worldwide participated in this session (Fig. 2).

**Fig. 2:**
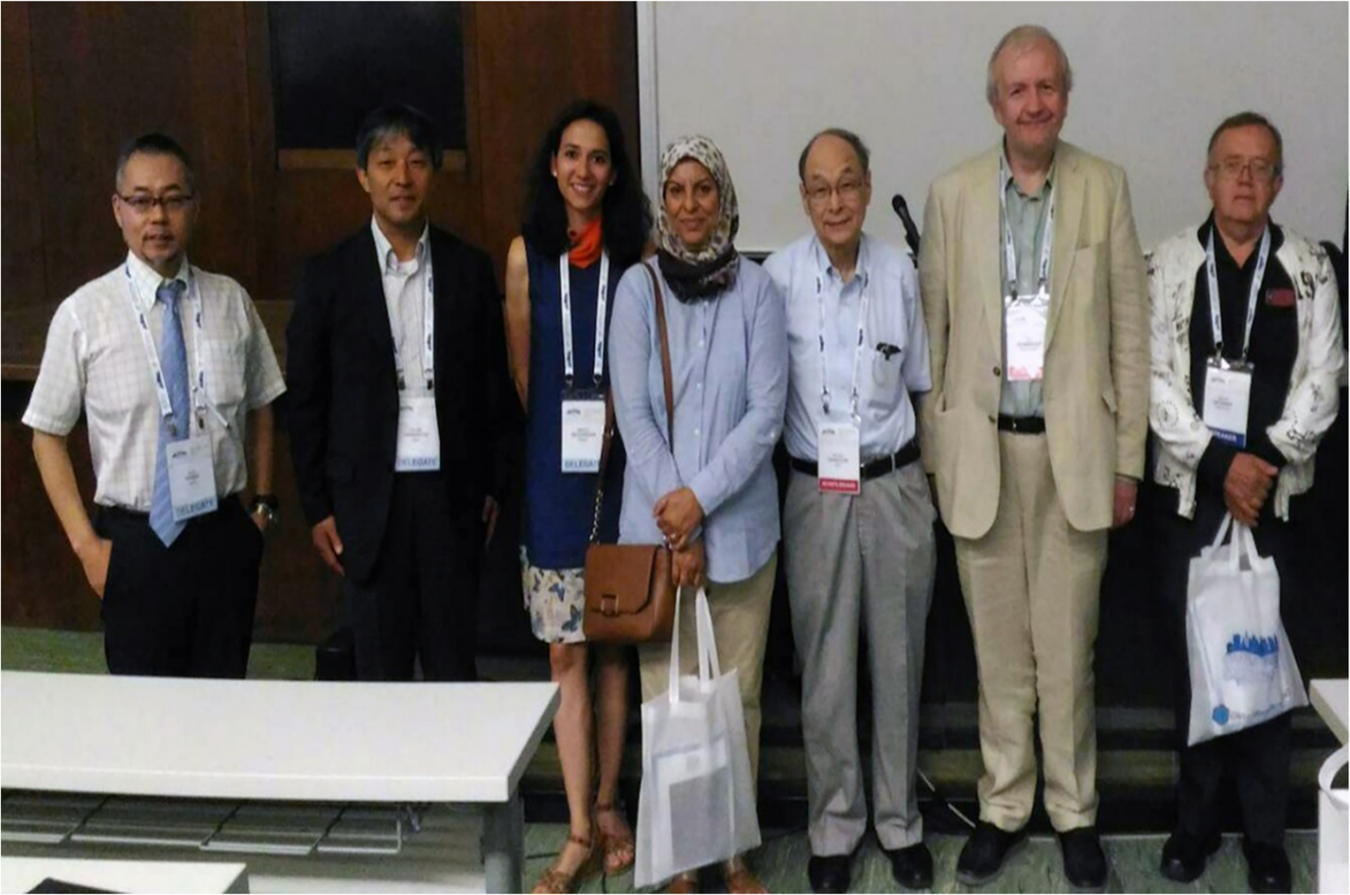
A group photo of main speakers and organizers taken at the session of glycation in Glyco25

In this issue Murata reports an update on browning and pigmentation in food through the Maillard reaction and other authors mainly focused on glycation in the medical sciences.

Main speakers and organizers at the session of glycation at Glyco25.
